# Supplementary figures and images for: Mechanisms of Zero-Lag Synchronization in Cortical Motifs
Source: PLoS Comput Biol. 2014 Apr 24;10(4):e1003548. doi: 10.1371/journal.pcbi.1003548 (PMC3998884; doi:10.1371/journal.pcbi.1003548)

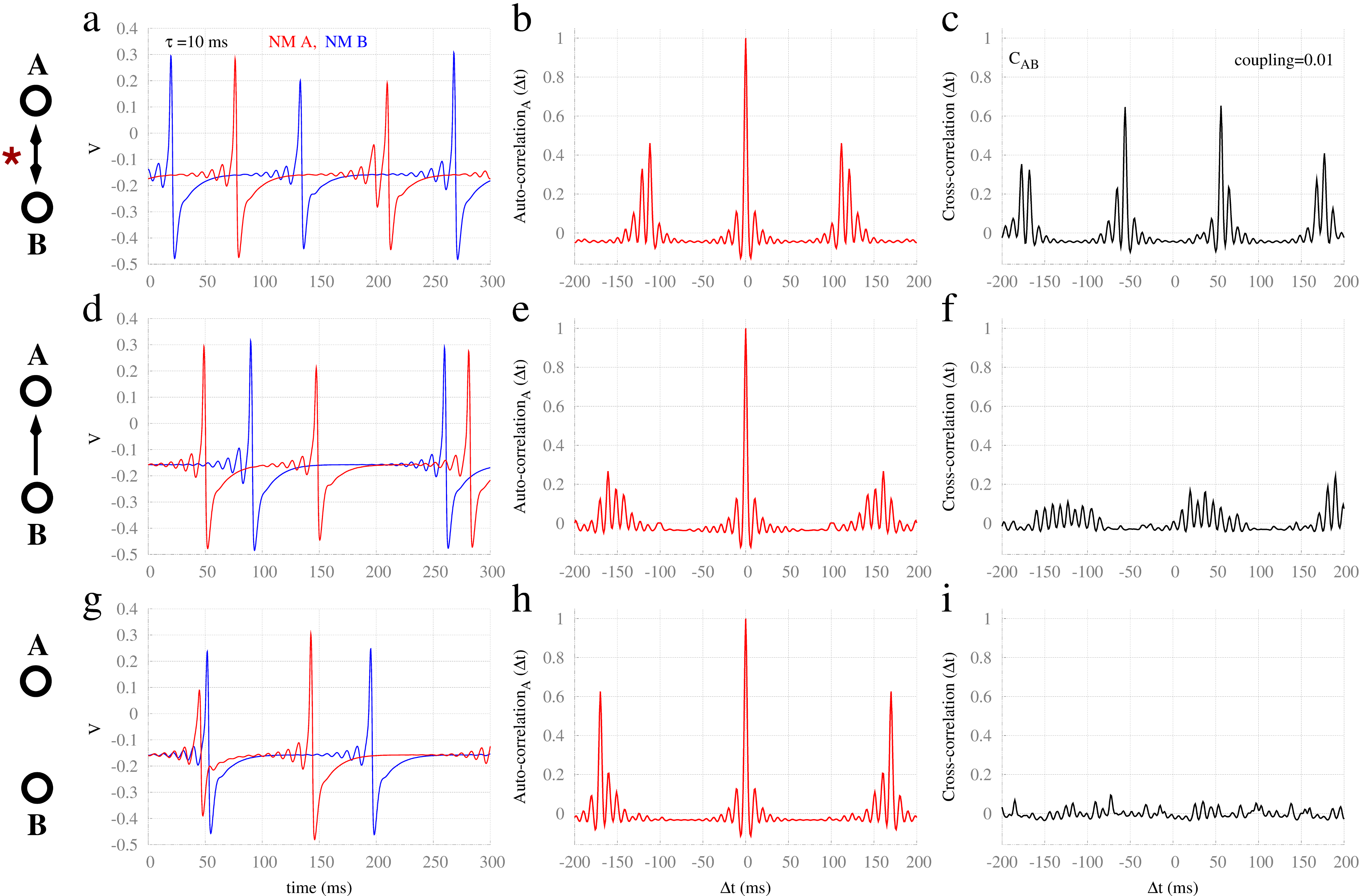

Supplement: Figure S1 — Dynamics of pairs of neural mass models. (a), (d) and (g) show the time traces of the average membrane potential of the excitatory pyramidal neurons; (b), (e) and (h) show the auto-correlation function of node A; (c), (f) and (i) show the cross-correlation function between nodes A and B; respectively for a pair bidirectionally connected, unidirectionally connected, and disconnected nodes. Parameters are c = 0.01, and . (TIFF) [file pcbi.1003548.s001.tiff]

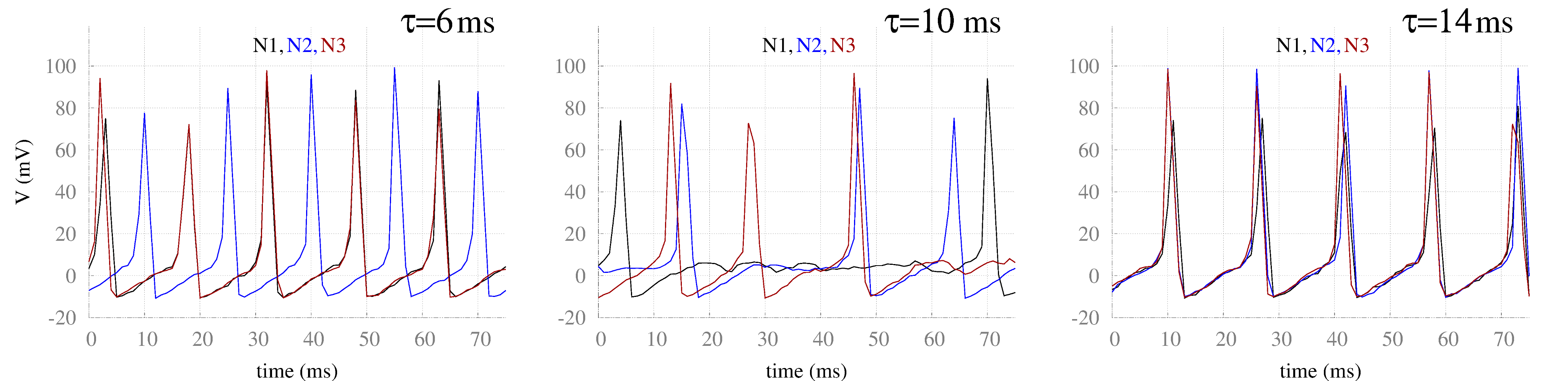

Supplement: Figure S2 — Example dynamics of Hodgkin-Huxley neurons coupled on motif M6 for different time delays. From left to right, panels show anti-phase synchronization (), no synchronization (), and phase synchronization (). (TIFF) [file pcbi.1003548.s002.tiff]

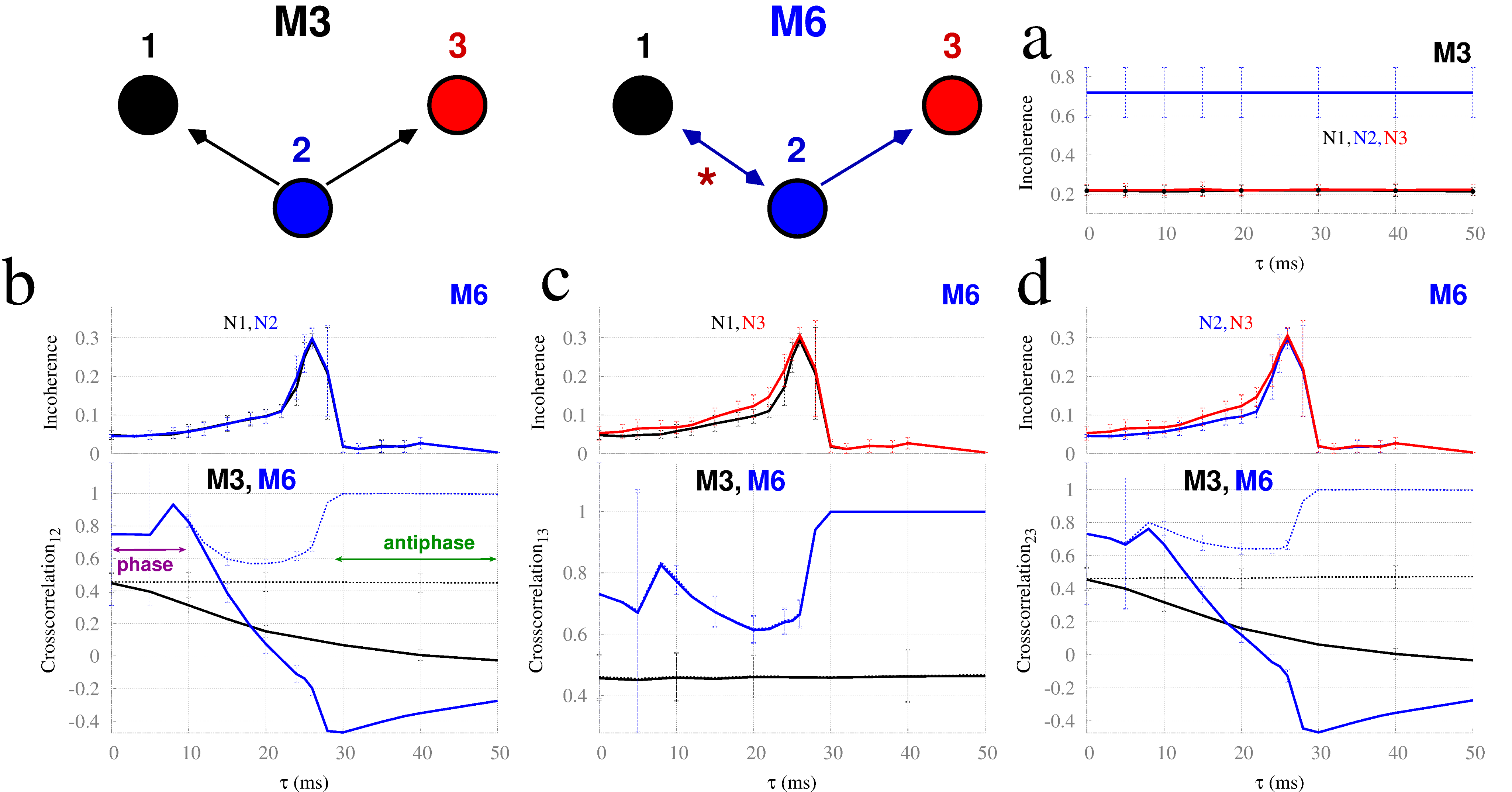

Supplement: Figure S3 — Synchronization dynamics and incoherence in populations of Izhikevich neurons. Panels (a–d) as per Fig. 3 but for populations of spiking neurons. Phase, anti-phase synchrony, and a state of phase synchrony at the slow rhythm and anti-phase synchrony at the fast rhythm can be found in motif M6 depending on the time delay (see exemplar time traces in supplementary Fig. S4). (TIFF) [file pcbi.1003548.s003.tiff]

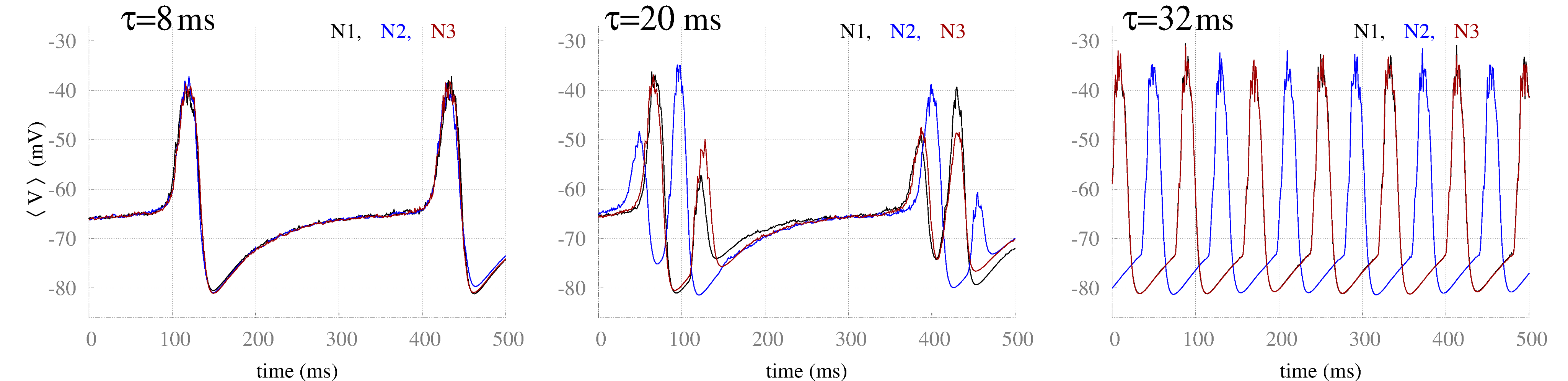

Supplement: Figure S4 — Example dynamics of populations of Izhikevich neurons coupled as motif M6 for different time delays. From left to right, panels show phase synchronization (), phase synchronization at the slow rhythm and anti-phase synchronization at the fast rhythm (), and anti-phase synchronization (). (TIFF) [file pcbi.1003548.s004.tiff]

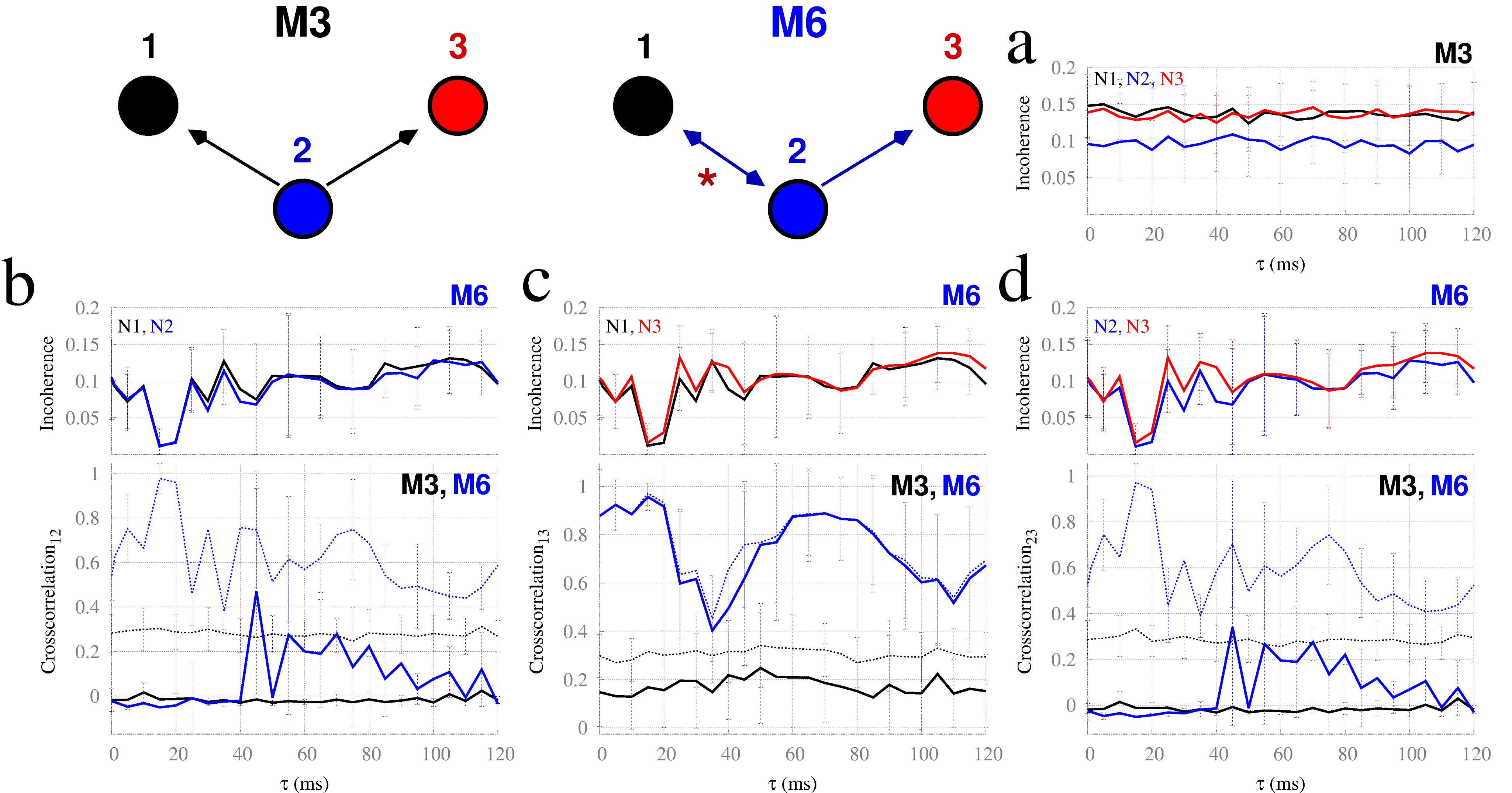

Supplement: Figure S5 — Synchronization dynamics and incoherence in weakly coupled neural mass models. Panels (a–d) as per Fig. 3 but for neural mass models with coupling strength c = 0.01. Anti-phase synchrony at the slow or at the fast rhythms, and a state of low synchrony can be found in motif M6 depending on the time delay (see exemplar time traces in supplementary Fig. S6). (TIFF) [file pcbi.1003548.s005.tiff]

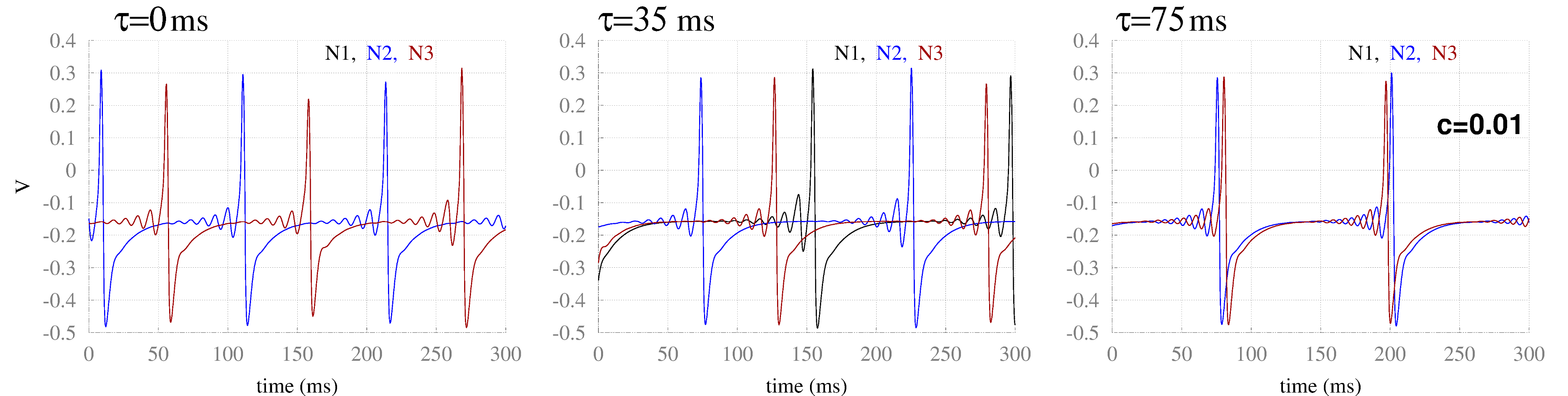

Supplement: Figure S6 — Example dynamics of neural mass models coupled on motif M6. From left to right, panels show anti-phase synchrony at the slow rhythm (no time delay), weak synchrony (), and anti-phase synchrony at the fast timescale (). The coupling strength is weak, c = 0.01. (TIFF) [file pcbi.1003548.s006.tiff]

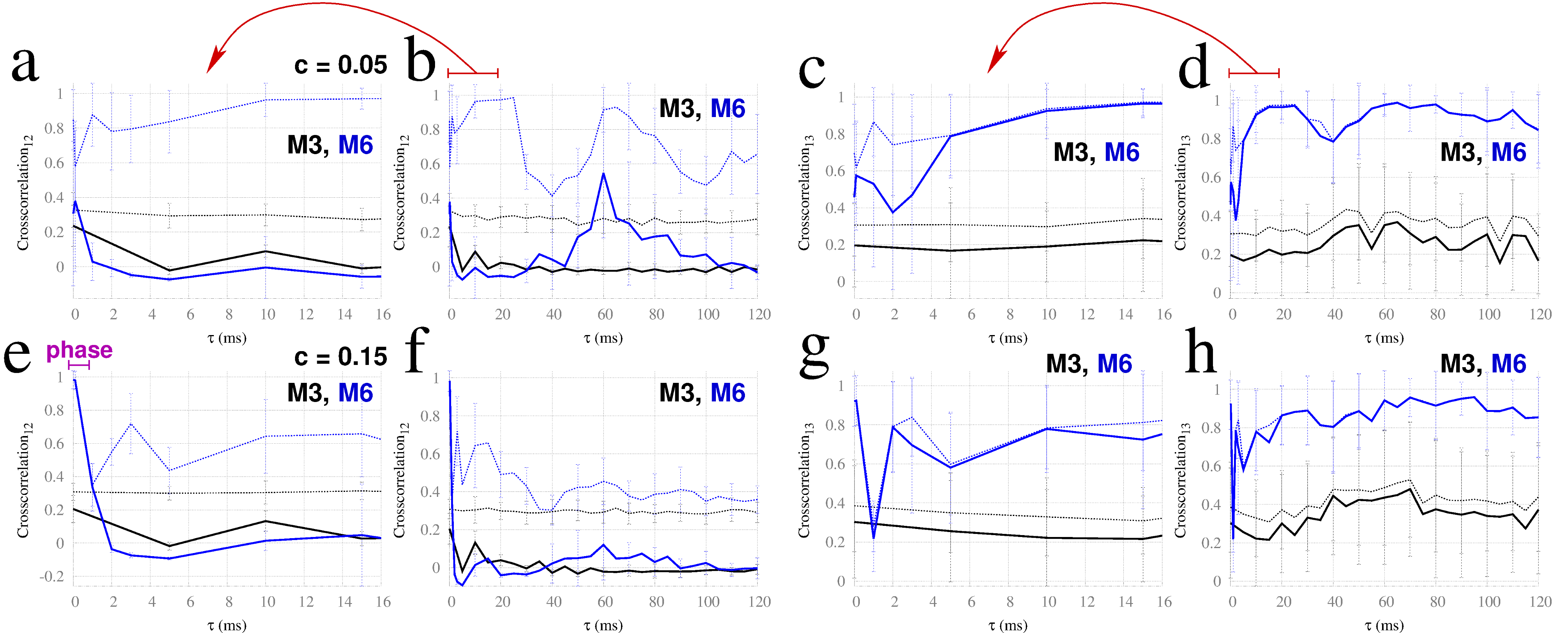

Supplement: Figure S7 — Cross-correlation for strongly coupled neural mass models. Top panels show the crosscorrelations between nodes 1 and 2 (a–b), and nodes 1 and 3 (c–d) as a function of the delay for coupling strength c = 0.05. Bottom panels panels show the cross-correlations between nodes 1 and 2 (e–f), and nodes 1 and 3 (g–h) as a function of the delay for coupling strength c = 0.15. First and third columns correspond to a zoom of second and forth columns respectively. Black (blue) lines represent results for motif M3 (M6), and continuous (dashed) lines represent the crosscorrelation at zero lag (maximum for all time lags). Phase synchrony, and complex synchronous states can be found in motif M6 depending on the time delay (see exemplar time traces in supplementary Fig. S8). Results are averaged over 40 trials. (TIFF) [file pcbi.1003548.s007.tiff]

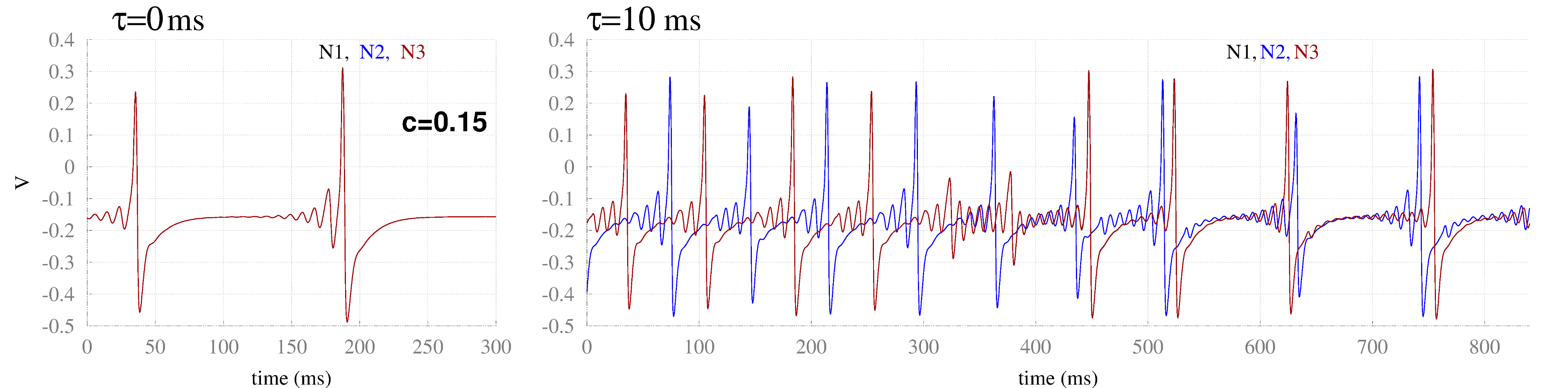

Supplement: Figure S8 — Example dynamics of neural mass models strongly coupled as motif M6 for different time delays. From left to right, panels show phase synchrony (), and a transition from a state of anti-phase synchrony at the slow rhythm to a state of out-of-phase synchrony (). The coupling strength is c = 0.15. (TIFF) [file pcbi.1003548.s008.tiff]

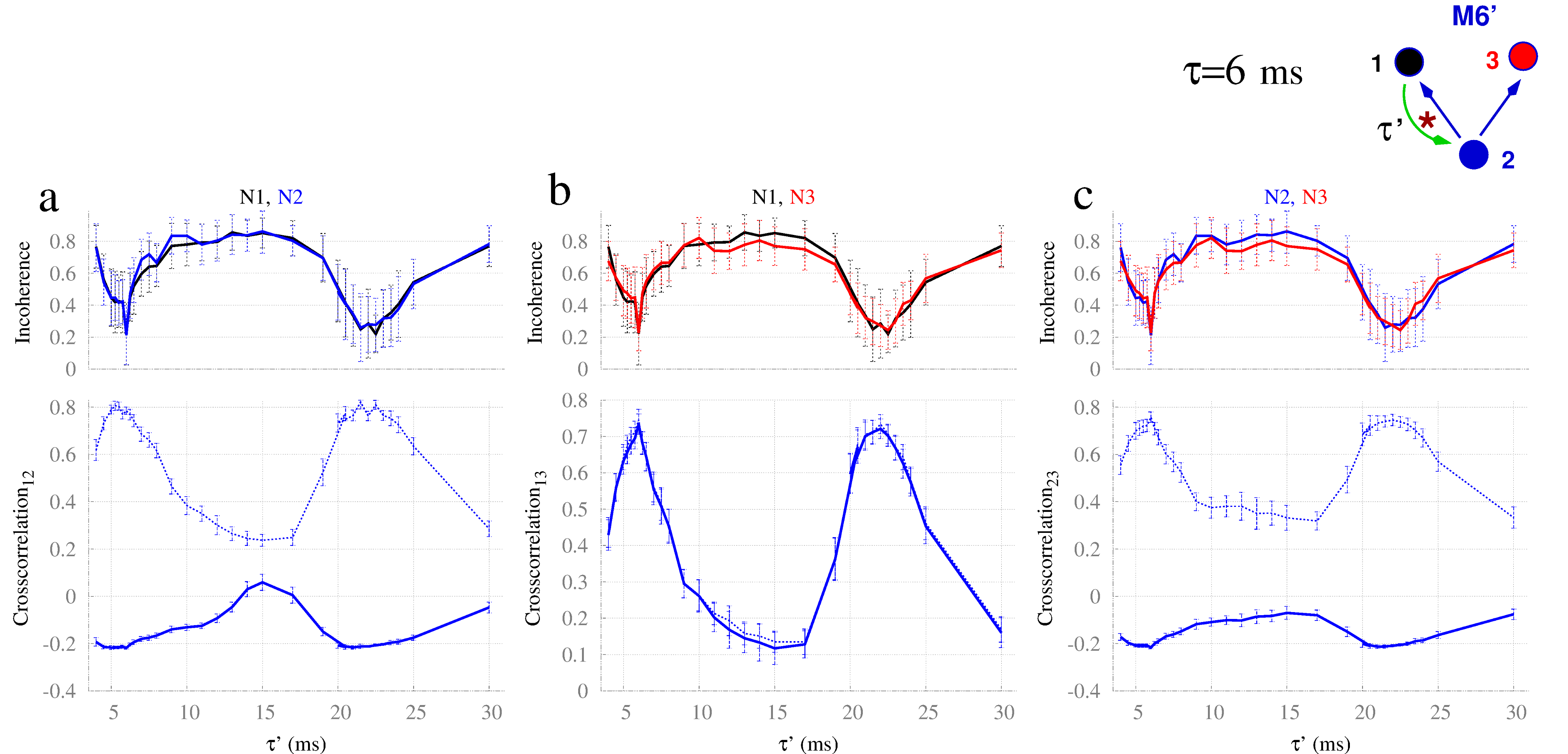

Supplement: Figure S9 — Incoherence and synchronization dependence on the time-delay mismatch in Hodgkin-Huxley neurons. (a–c) Top panels show incoherence: Colors represent different nodes. Bottom panels show cross-correlations for motif M6′. Continuous lines indicate the cross-correlation coefficients at zero time lag, and dashed lines indicate the maximum cross-correlation coefficients across all time lags. Panels a, b and c represent pairs of nodes: 1–2, 1–3, and 2–3 respectively. Results are averaged over 40 trials. (TIFF) [file pcbi.1003548.s009.tiff]

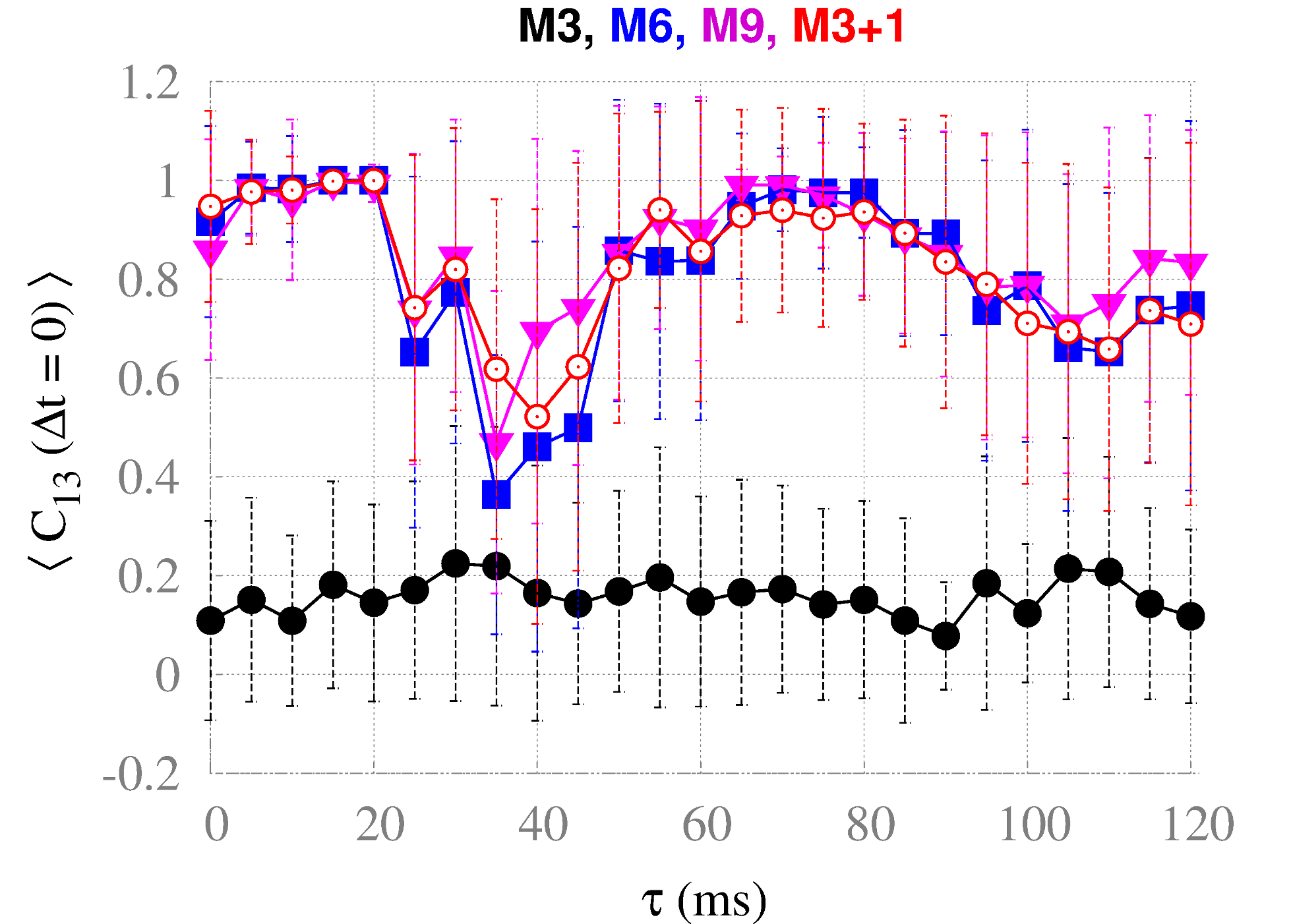

Supplement: Figure S10 — Zero-lag synchronization dependence on the delay in motifs of neural mass models. The curves are color coded as in Fig. 7. In agreement with [40] the synchronization depends on the coupling delay for long delays. The coupling strength is c = 0.01. Crosscorrelation is averaged over 40 trials. (TIFF) [file pcbi.1003548.s010.tiff]

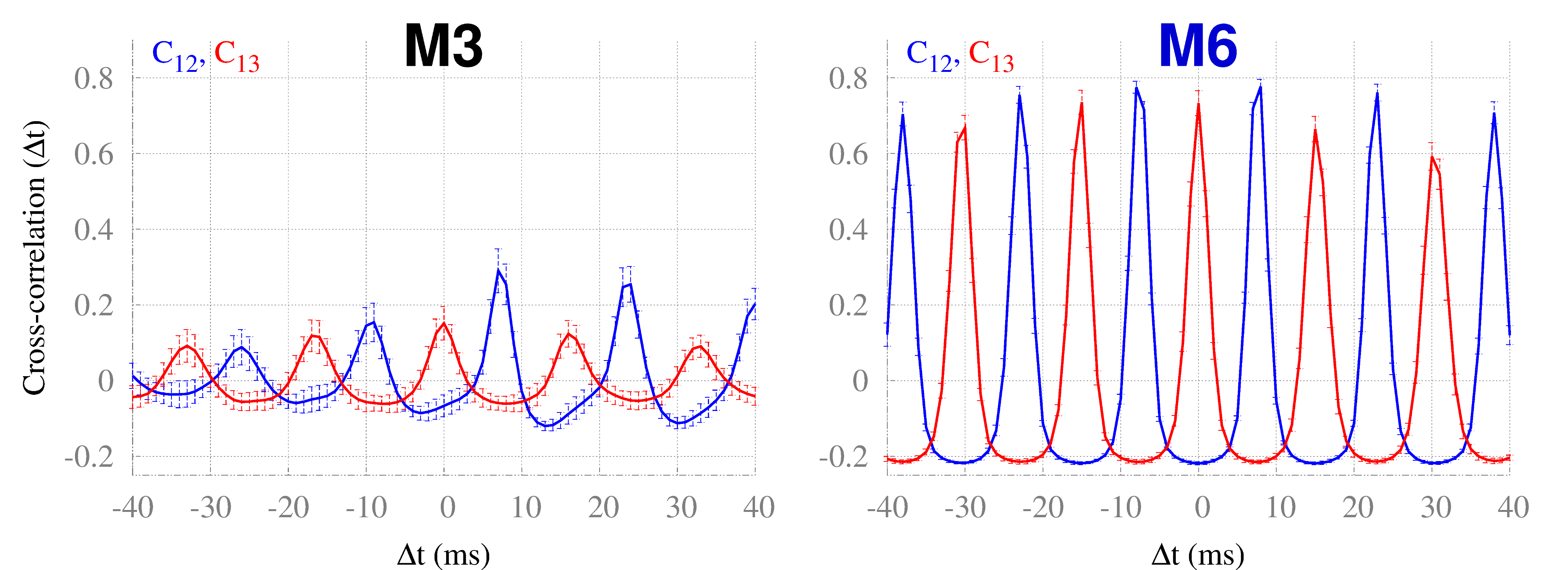

Supplement: Figure S11 — Kernel test in motifs of Hodgkin-Huxley neurons. Nearly identical cross-correlation functions are obtained when the external driving is considered identical to the spikes within the motifs (see Fig. 2, panels d and h). Plot corresponds to an average over 40 trials. (TIFF) [file pcbi.1003548.s011.tiff]
